# An estimate of the prevalence of dementia in Africa: A systematic analysis

**DOI:** 10.7189/jogh.02.020401

**Published:** 2012-12

**Authors:** Rhiannon George-Carey, Davies Adeloye, Kit Yee Chan, Abigail Paul, Ivana Kolčić, Harry Campbell, Igor Rudan

**Affiliations:** 1 Centre for Population Health Sciences, University of Edinburgh Medical School, Edinburgh, Scotland, UK; 2 Nossal Institute for Global Health, Melbourne University, Melbourne, Australia; 3 Department of Health Policy and Management, School of Public Health, Peking University Health Science Centre, Beijing, China; 4 Croatian Centre for Global Health, University of Split School of Medicine, Split, Croatia

## Abstract

**Background:**

The burden of non–communicable diseases is growing, particularly in developing countries. The greatest economic burden is due to dementia, the prevalence of which is rising with increasing longevity. In Africa, where the rate of increase of elderly persons is the fastest in the world, dementia is normally dismissed as a part of normal ageing. The lack of awareness means that many patients are suffering undiagnosed. This review aims to assess the information on the prevalence of dementia in Africa in order to estimate the current burden.

**Methods:**

A parallel search of Medline, EMBASE and Global Health limited to post–1980 found only 10 relevant studies. Data on prevalence and risk factors were extracted and analysed. We modelled the available information and used the UN population figures for Africa to determine the age–specific and overall burden of dementia.

**Results:**

The overall prevalence of dementia in adults older than 50 years in Africa was estimated to be about 2.4%, which translates to 2.76 million people living with a disease in 2010. About 2.10 millions of them live in Sub–Saharan Africa. Prevalence was the highest among females aged 80 and over (19.7%) and there was little variation between regions. Alzheimer disease was the most prevalent cause of dementia (57.1%) followed by vascular dementia (26.9%). The main risk factors were increasing age, female sex and cardiovascular disease.

**Conclusions:**

Information on dementia prevalence in Africa is very limited. Further research will not only provide a more reliable estimate of prevalence, and consequently the burden of disease, but will also raise awareness of the problem. This is critical in promoting help–seeking behaviour and generating the political commitment to make dementia a public health priority in Africa.

Non–communicable diseases (NCDs) have become the leading causes of death globally [1]. NCDs kill more people than all other diseases combined, accounting for 63% of deaths in 2008 [2,3]. It is often under–appreciated that nearly 80% of these deaths occur in low– and middle–income countries (LMICs), where ageing is presently occurring at a faster rate [1]. From 1950 to 2009, 68% of the increase in persons aged 60 or over was seen in LMIC [4].

This is true even for African nations, where the average life expectancy is still only 55.2 years, due to high mortality from communicable, maternal, perinatal and nutritional causes [5]. The current African context is described in more detail in **Box 1** while **Box 2** briefly describes the characteristics of the disease of interest [5,6].

Box 1 **A summary of the current African context**

In Sub–Saharan Africa nearly 6% of adults aged 15–49 are HIV positive, and malaria kills a million children under the age of five each year, with childhood pneumonia, diarrhoea and other infectious diseases still being a major cause of mortality [6]. Nevertheless, non–communicable diseases (NCDs) are expected to exceed them all as the most common cause of death by 2030 [1].

Middle class population of Africa has tripled over the last thirty years to become more than 34% of the continent’s population [7]. This growth means more people will seek better health care for themselves and their families and this is likely to contribute to patterns of morbidity and mortality. As its population is ageing faster than that in high–income countries, the necessity of prevalence data from Africa must be emphasised [8]. The health of the elderly is of special importance in this context, as they are filling the roles of the generation decimated by HIV/AIDS, particularly in Sub–Saharan Africa [9]. Only a few community–based studies have been carried out in Africa and these have often reported a low prevalence of dementia [10]. Suggested reasons for this include: (i) poor access or reluctance to seek medical care; (ii) the belief that an elderly person has completed their useful life; (iii) differential survival rates; (iv) hiding of cases by relatives concerned about the stigma of mental disease; (v) defective case finding techniques; and (vi) the belief that dementia is a normal part of ageing [11].

Without appropriate and sufficient knowledge on the burden of dementia in Africa, this disease will not be considered a public health priority, making it difficult to secure resources necessary to improve the management of patients [12]. However, the recent report by the World Health Organization stated that “...there is no more powerful tool for obtaining political and financial commitment than locally derived and relevant data” [13]. A reliable estimate of the prevalence could therefore considerably advance further research into the burden, as well as increase understanding of dementia in Africa and low– and middle– income countries (LMICs) in general.

Box 2 **A summary of background information on dementia**

Dementia is a syndrome, usually of a chronic or progressive nature, in which there is deterioration in cognitive function. This deterioration can be caused by a number of brain disorders or injuries that primarily or secondarily affect the brain, such as Alzheimer disease or stroke. Dementia affects higher cortical functions such as memory, thinking, comprehension and judgment. This impairment is commonly accompanied by a decline in emotional control, social behaviour, or motivation [13]. Diagnosis is conventionally made when cognitive decline affects a person’s ability to carry out daily routine activities [14]. Many different forms or causes of dementia exist. Alzheimer disease is the most common, contributing to 60–70% of cases. It is characterised by cortical amyloid plaques and neurofibrillary tangles, and symptoms include a gradual onset of impaired memory, apathy and depression [14]. Early–onset Alzheimer disease can present before the age of 65 and normally has a genetic cause [15]. Vascular dementia accounts for 20–30% of dementia [14]. It is diagnosed when the brain’s oxygen supply is compromised by repeated strokes or other blood vessel pathology, leading to accumulated damage to brain tissue and function [15]. Symptoms are similar to those of Alzheimer disease although memory is less affected and mood fluctuations are more [14]. Other common subtypes are frontotemporal dementia (5–10%) and Lewy body dementia (<5%). Post–mortem studies have suggested that boundaries between different forms are indistinct and subtypes often co–exist [14]. Dementia is largely under–diagnosed and often by the time of diagnosis patients are at a late stage in the disease process [16]. This is a particular issue in low and middle income countries (LMICs), as families may not understand their relative’s behaviour [14]. Early diagnosis is helpful so that everyone involved can be better equipped to deal with the disease and know what to expect [15].

Risk factors

The main risk factor for dementia is advancing age, and after the age of 65 the prevalence doubles every five years [17]. The environment is thought to influence the development of the disease, although little is understood about the underlying mechanisms [15]. Other suspected risk factors include: (i) cardiovascular problems; (ii) excessive alcohol consumption; (iii) social isolation; (iv) head injury; and (v) having one or two copies of the APOEϵ4 genetic variant. However, moderate alcohol consumption and oestrogen have been reported to reduce the risk of developing dementia [18].

Course and outcome

Dementia is usually progressive and irreversible unless a cause is identified and treated effectively. Each person is affected in a different way, with varying speed of deterioration. Generally, the lifespan of the affected person is reduced [15]. Disease course is normally characterised into three stages: early, middle and late. The early stage is often overlooked as ‘old age’. However, as the disease progresses into the middle stage limitations become clearer and more restricting. By the late stage, the person affected becomes nearly entirely dependent, with severe memory disturbances and physical symptoms [14].

Management

Currently, no treatments to cure or alter the progression of dementia exist. Patients can be treated symptomatically with pharmacological or psychosocial interventions. The latter are often highly effective and should be the first choice when managing behavioural problems [15]. Moreover, the provision of information and support has been shown to reduce the psychological distress often experienced by carers [15].

Resources and prevention

Primary preventive intervention is a highly cost–effective, yet neglected area. Effective interventions could significantly reduce dementia prevalence and incidence, improve the quality of life of patients and carers, and reduce the resources needed to provide adequate institutional and home health care [19].

NCDs are not only a health problem, but also a social problem, significantly contributing to poverty. Most NCDs are chronic and lead to continued expenditures that can trap households in cycles of debt and illness [20]. They tend to disproportionately affect poorer individuals, largely due to inequalities in the distribution of major risk factors [20]. The UN's High–Level Meeting in September 2011 put the global pandemic of NCDs at the top of the global political agenda [9,21]. Gross disparities in resources exist within and between developed and developing countries: new drug treatments are expensive; access to care is often dependent upon sufferers' means to pay; and health care resources are unevenly distributed between rural and urban districts [13-17]. Health services in LMICs are ill–equipped to meet the needs of older persons, as health care is primarily clinic–based, with treatments oriented towards acute rather than chronic conditions [2].

One of the NCDs expected to pose the greatest problem to health systems in LMICs in the coming decades is dementia. It has a uniquely profound effect on disability and need for care, being the major cause of dependency among the elderly worldwide [13,18]. Dementia affects not only individuals but also their families on personal, emotional, social and financial levels, driving millions of households below the poverty line and making dementia a global health priority [14-17,19]. It has been estimated 35.6 million people suffer from it worldwide – 0.5% of the world’s total population – and this is set to increase to over 115 million by 2050 [13,19]. Slightly more than half of the current sufferers live in LMICs, with a predicted increase of this figure to 71% by 2050 if the present trends continue [22]. The global economic impact of dementia for 2010 was estimated to amount to approximately US$ 600 billion, corresponding to 1.0% of the aggregated worldwide GDP, but per capita costs varied from US$ 868 in low–income countries, US$ 3109 in middle–income countries, to US$ 32685 in high–income countries [1].

As a part of international response to dementia, the WHO launched the Mental Health Gap Action Programme in 2008, with dementia as a priority condition, and it recently published a large report “Dementia: A public health priority” [1,13,15]. The group Alzheimer Disease International keeps publishing the “World Alzheimer's Report” annually, as a laudable attempt to keep track on the burden and impact of the condition [14,16,17]. Finally, in 2011, the High–level Meeting of the United Nations General Assembly on prevention and control of NCDs acknowledged that “mental and neurological disorders, including Alzheimer’s disease, are an important cause of morbidity and contribute to the global non–communicable disease burden” [13]. Despite these, addressing the problem of dementia remains a low priority in most LMICs, which tend to prioritise control and eradication of communicable diseases and reproductive, maternal, and child health. This is not surprising because even in high–income countries the prioritised NCDs are mainly those that cause early death, such as cancer and heart disease, and not those that cause years–lived–with–disability (YLD), such as dementia [7,23].

The objective of this paper is to systematically review publically available epidemiological studies on dementia and its main forms (Alzheimer disease and vascular dementia) in Africa and to estimate the burden of dementia in Africa in 2010. It also investigates differences in prevalence by age and gender, to highlight the segments of the population requiring the most urgent attention. The paper also aims to suggest policy implications and to contribute to an improvement in evidence and information on dementia in LMICs.

## METHODS

### Definitions and study design considerations

The chronic nature of dementia makes an estimate of prevalence more suitable than one of incidence [17]. Prevalence here refers to people aged 50 years and over with dementia in 2010. This age cut–off value (50+) was introduced because 50 years was the lowest age of participants included in the studies in this review.

### Search strategy

After initial scoping exercises to identify key words and Medical Subject Headings (MeSH) and input from a librarian to choose the final search terms (**Table 1**), a systematic review of published literature was undertaken across the following databases via OVID:

**Table 1:** Search terms

1.	Prevalence/ or incidence/ or morbidity/ or mortality/
2.	(prevalen* or inciden*).mp.
3.	(disease adj3 burden*).mp.
4.	1 or 2 or 3
5.	Dementia.tw.
6.	Alzheimer*.tw.
7.	5 or 6
8.	Exp Africa/ or exp africa, northern/ or exp algeria/ or exp egypt/ or exp libya/ or exp morocco/ or exp tunisia/ or “africa south of the sahara”/ or africa, central/ or exp cameroon/ or exp central african republic/ or exp chad/ or exp congo/ or exp “democratic republic of the congo”/ or equatorial guinea/ or exp gabon/ or exp africa, eastern/ or exp burundi/ or exp djibouti/ or exp eritrea/ or exp ethiopia/ or exp kenya/ or exp rwanda/ or exp somalia/ or exp sudan/ or exp tanzania/ or exp uganda/ or exp africa, southern/ or exp angola/ or exp botswana/ or exp lesotho/ or exp malawi/ or exp mozambique/ or exp namibia/ or exp south africa/ or exp swaziland/ or exp zambia/ or exp zimbabwe/ or exp africa, western/ or exp benin/ or exp burkina faso/ or exp cape verde/ or exp cote d'ivoire/ or exp gambia/ or exp ghana/ or exp guinea/ or exp guinea-bissau/ or exp liberia/ or exp mali/ or exp mauritania/ or exp niger/ or exp nigeria/ or exp senegal/ or exp sierra leone/ or exp togo/
9.	4 and 7 and 8
10.	Limit 9 to (humans and yr = ”1980-Current”)

1) Medline (1980–Current) on 22 January 2012;

2) EMBASE (1980–Current) on 9 February 2012;

3) Global Health (1980–Current) on 9 February 2012.

An additional search of Google Scholar and the hand–searching of the selected studies’ reference lists produced no additional results. Specific countries were those identified by the World Bank list of economies (January 2012) as being LMICs in Africa. Limitations were placed to studies concerning humans and those published after 1980.

### Study selection

**Figure 1:**
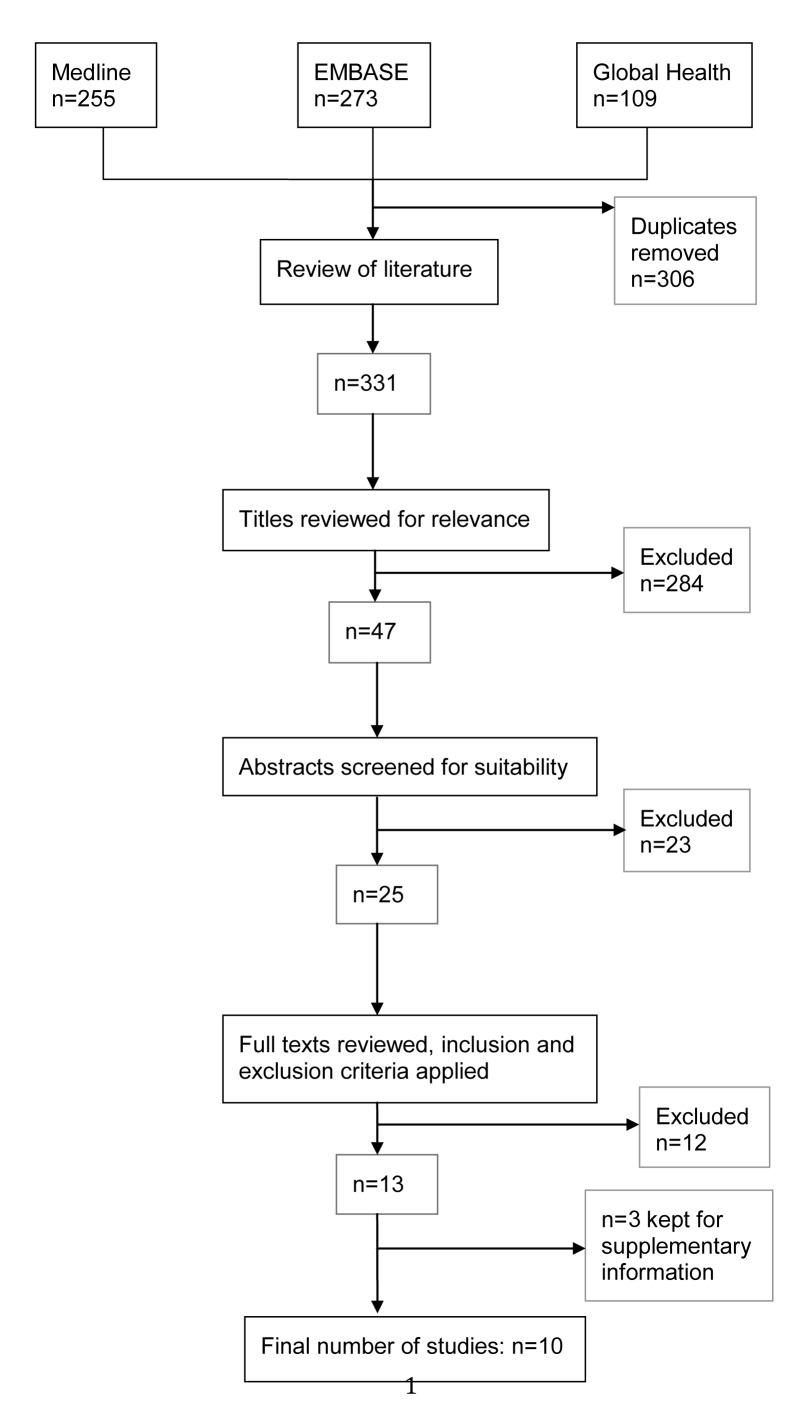
A summary of search strategy used to identify the studies relevant to estimating the prevalence of dementia in Africa.

**Figure 2:**
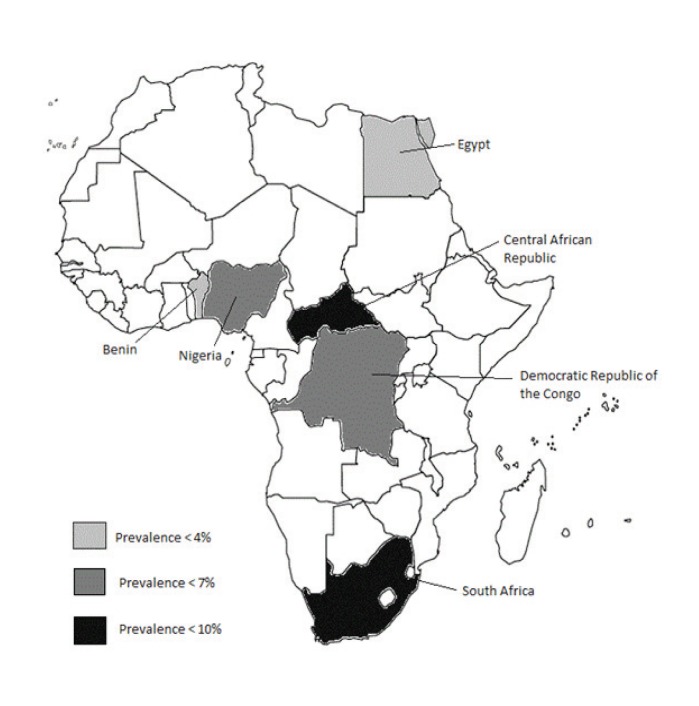
Geographic locations of retained studies with overall prevalence estimates; most studies were active during 2002–2009.

The inclusion criteria used to screen the useful papers were: (i) community–based studies; (ii) conducted in LMICs in Africa, any age–group or sex; (iii) reporting prevalence of dementia. We excluded all studies that were either hospital–based, published before 1980, studies without a numerical estimate for prevalence, studies with subjects not human, or review articles (see **Figure 1**). We then evaluated the retained studies for quality of their design and methods. We required a clear case definition of dementia, with a defined nominator, denominator and time frame of the study, appropriate study design for a prevalence study, and adequate explanation of processes undertaken, including recruitment of subjects (see **Figure 2** and **Table 2**). Case definitions were mostly similar, with considerable overlap between studies, and they should not have had a significant influence on the analyses (**Table 3**). Hospital–based studies were excluded; it cannot be presumed that everyone in LMIC has access to medical facilities and dementia may be secondary to another condition, for which the patient has been hospitalised.

**Table 2:** Characteristics of studies included in the review

Characteristics	No. of studies	
**Country:**		
All		
Nigeria		4
Egypt		2
Benin		2
South Africa		1
Central African Republic		1
Republic of the Congo		1

Duration of study:	
<1 year	5
1–2 years	4
3–4 years	1

Sample size:	
0–1000	4
1001–2000	3
2001–3000	2
>3000	1

Setting:	
Urban	6
Rural	2
Urban and rural	2

**Table 3:** Case definitions and screening measures of dementia in retained studies

Author	Country	Case definition	Screening measures	Complications
Ben-Arie (1983) [24]	South Africa	n/a	MMSE, PSE, FCA	Life Satisfaction Index Social Performance Schedule
El Tallaway (2010) [25]	Egypt	n/a	SSQ, FCA	Other neurological conditions
Farrag (1998) [26]	Egypt	DSM–III–R NINCDS–ADRDA Hachinski Ischaemic Score	SSQ, MMSE, FCA, laboratory diagnosis	IADL Geriatric Depression Scale Revised Weschler Adult Intelligence Scale
Guerchet (2009) [27]	Benin	DSM–IV NINCDS–ADRDA	SSQ, CSI–D, FCA	Goldberg’s Anxiety and Depression Scale Jacoby’s Stigma Scale Psychosocial factors according to Persson and Skoog Medical examination
Guerchet (2010) [28]	Central Republic of Africa Republic of the Congo	DSM–IV NINCDS–ADRDA Hachinski Ischaemic Scale	SSQ, CSI–D, 5WT, FCA	Medical history Psychosocial factors according to Persson and Skoog Goldberg’s Anxiety and Depression Scale
Gureje (2006) [29]	Nigeria	DSM–IV	10WDRT	World Mental Health Survey version of WHO Composite International Diagnostic Interview Katz Index of Independence of Daily Living Nagi Physical Performance Scale and Health Assessment Questionnaire WHO–QOL–BREF
Hendrie (1995) [30]	Nigeria	DSM–III–R ICD–10	SSQ, CSI–D, CERAD	n/a
Ochayi (2006) [11]	Nigeria	DSM–IV ICD–10	CSI–D	Medical examination
Paraiso (2011) [12]	Benin	DSM–IV NINCDS–ADRDA NINDS–AIREN	SSQ, CSI–D, FCA	Medical examination Goldberg’s Anxiety and Depression Scale Psychosocial factors according to Persson and Skoog
Yusuf (2011) [31]	Egypt	DSM–IV ICD–10	SSQ, CSI–D, CERAD, SDT, BDS	n/a

DSM – Diagnostic and Statistical Manual of Mental Disorders, NINCDS–ADRDA – National Institute of Neurological and Communicative Disorders and Stroke – Alzheimer Disease and Related Disorders Association, ICD–10 – International Classification of Diseases – 10, NINDS–AIREN – National Institute of Neurological Disorders and Stroke – Association Internationale pour la Recherché et l'Enseignement en Neurosciences, MMSE – Mini Mental State Examination, PSE – portal–systemic shunt encephalopahty, FCA – Family Caregiver Alliance, SSQ – Speech, Spatial and Qualities of Hearing Scale, CSI–D – CommunityScreening Interview for Dementia, 5WT – Five Word Test, 10WDRT – 10–Word Delay Recall Test, CERAD – Consortium to Establish a Registry for Alzheimer Disease, SDT – sibship disequilibrium test, BDS – Blessed Dementia Scale, IADL – Instrumental Activities of Daily Living

### Data extraction

Study characteristics and other relevant data were extracted from each retained article and entered into Microsoft Excel spreadsheet. Noted study characteristics include: country, region, period of study, setting, sample, age of participants, population characteristics, study size and type of assessor (see Online Supplementary Document(Online Supplementary Document)). Wherever more than one article referred to the same study site / cohort, the first chronologically published paper was entered into the database and all additional data from other papers were added to the initial study (see Online Supplementary Document(Online Supplementary Document) for further details). All data on dementia prevalence and the prevalence of its two most important subtypes (Alzheimer disease and vascular dementia) were extracted: the total sample size for each study, number of cases with disease, and the prevalence in the overall sample. In addition, we extracted each prevalence which was reported within a specific age–group, whether it was disaggregated by gender or not (see Online Supplementary Document(Online Supplementary Document)).

### Data analysis

**Figure 3:**
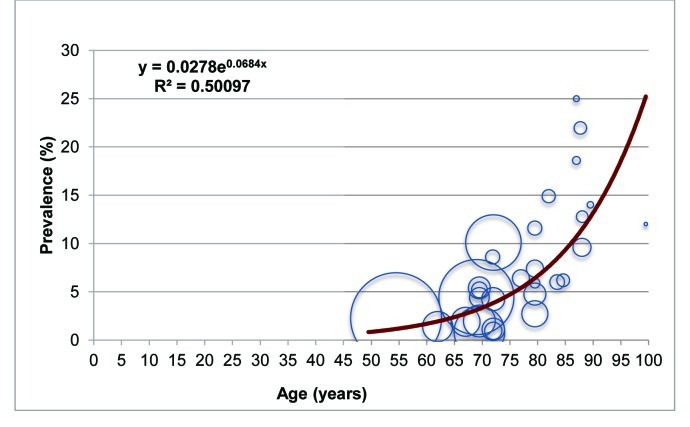
Age–specific prevalence of dementia in Africa based on the information from 10 studies, with sample size underlying each data point presented by the size of each bubble.

Our aim was to establish the pattern of relationship between person's age and likelihood to suffer from dementia (or its two subtypes) across the age range starting from 50 years of age until death, where the overwhelming majority of cases (>99%) are expected to occur in the population. This relationship was modelled based on the available information from all retained studies. All the extracted information from individual studies was plotted in a graph defined by age (x–axis) and prevalence of dementia (or its 2 subtypes; y–axis). The coordinate on x–axis was defined as the mean age of the persons included in each (sub)sample, while the coordinate on y–axis was defined by the reported prevalence in that (sub)sample. The size of the “bubble” plotted in the graph was proportional to sample size relevant to each individual source of information. For all age groups that were not fully defined, such as eg, “60+ years”, mean age was determined as the age that comprised 50% of the population in this age group in the country where the study was undertaken, according to the UN Population Division's estimates for the year of study. Graphs were then plotted with mean age on x–axis and dementia prevalence on y–axis, taking into account sample sizes which were represented by circle size (see eg, **Figure 3**). Graphs were drawn for both dementia and its two most common forms – Alzheimer disease and vascular dementia; for dementia, two additional analysis were possible – for men and women.

Given that the lowest mean age for any (sub)sample defined in the retained studies was 54.5, we defined the age group of 0–54 years as the one where no data were available, and assumed that the prevalence of dementia in that age group was close to zero. Thereafter, the information on the prevalence was organised by 5–year age groups (from 55–59 years; 65–74 years; 75–84 years; and 85 years and over). The weighted mean prevalence and standard deviation were then calculated for each of those four age–groups for dementia (**Table 4**), Alzheimer disease (**Table 5**) and vascular dementia (**Table 6**). Weighted mean prevalence was calculated as an average of the prevalence multiplied by sample size, while 95% confidence intervals were calculated from the standard errors of prevalence values.

**Table 4:** Age–specific weighted mean prevalence (%) of dementia in Africa

Age group (years)	55–64	65–74	75–84	85+
No. of data points	2	12	8	8
Mean age (years)	58.25	70.12	80.00	88.92
Weighted mean prevalence and standard error (in %)	2.17 (0.84)	5.66 (1.69)	6.14 (2.72)	12.94 (3.71)

**Table 5:** Age–specific weighted mean prevalence (%) of Alzheimer disease in Africa

Age group (years)	55–64	65–74	75–84	85+
No. of data points	1	6	7	4
Mean age (years)	62	70.15	78.45	87.42
Weighted mean prevalence and standard error (in %)	0.8 (–)	2.11 (1.05)	3.27 (3.23)	10.99 (7.81)

**Table 6:** Age–specific weighted mean prevalence (%) of vascular dementia in Africa

Age group (years)	55–64	65–74	75–84	85+
No. of data points	1	5	7	3
Mean age (years)	62	70.28	78.42	87.23
Weighted mean prevalence and standard error (in %)	0.3 (–)	0.94 (0.47)	0.63 (0.86)	2.88 (2.14)

**Figure 4:**
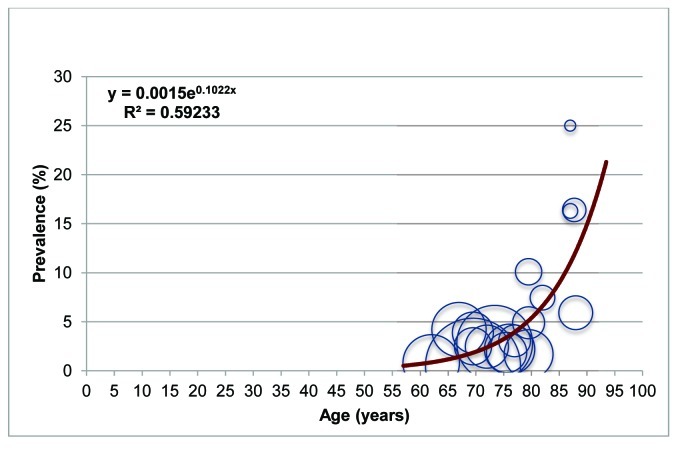
Age–specific prevalence of Alzheimer disease in Africa, with sample size underlying each data point presented by the size of each bubble.

**Figure 5:**
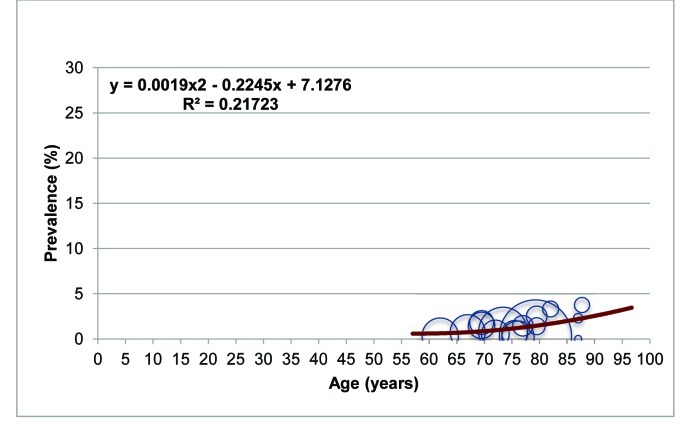
Age–specific prevalence of vascular dementia in Africa, with sample size underlying each data point presented by the size of each bubble.

To compute the overall burden of dementia, Alzheimer disease and vascular dementia in Africa, an epidemiological model was developed using meta–regression–like approach. A fundamental difference in this study was that the goal of this regression analysis was not to investigate the association between age and prevalence, but rather to use available data to predict the most likely prevalence at any given age after 50 years. The model with best “fit” (largest proportion of variance explained) was used for each disease (**Figures 3**, **4** and **5**). The equation of the trendline was then used to compute the predicted prevalence at the ages of 52, 57, 62, 67, 72, 77 and 87 years, which are mid–points of the UN Population Division's 5–year age group population estimates for Africa in 2010. We multiplied the predicted prevalence at each age with total population in the corresponding age groups to compute the total number of people living with dementia and its two main forms in Africa.

## RESULTS

### Systematic review

An initial screen of three databases returned 255 results from Medline, 273 results from EMBASE and 109 results from Global Health. After excluding duplicates, 331 titles were reviewed for relevance and only 47 studies were selected for review of abstract. Wherever the abstracts lacked a numerical estimate for prevalence, full texts were sourced. Eventually, only 10 studies were retained for the final review and data extraction [8,10-12,24,27,29-32]. The literature search process is outlined in **Figure 1**.

### Study characteristics

The majority of studies took place in Western Africa, with no studies conducted in Eastern Africa. The EDAC Survey found the prevalence of dementia in two Central African countries, the Central African Republic and the Republic of the Congo [8]. The clustering of studies in Nigeria could be attributed to the longitudinal study on the prevalence and incidence of dementia and Alzheimer disease in Yoruba people [30]. Half of the studies were conducted within less than one year. The mean sample size was 7263, but this is mainly a result of significant skewing due to one large study with 62 583 participants; the mean sample size of the other 9 studies was 1004. Studies were largely conducted in urban areas, with two of them conducted in both urban and rural settings. The majority of studies focused on prevalence in adults aged 65 years or more (**Table 2**).

### Case definitions

**Table 3** shows the case definitions and screening and complication measures used in each study. DSM and NINCDS–ADRDA were the most common criteria for Alzheimer disease diagnosis. ICD–10 and Hachinski Ishaemic Scale were used to diagnose vascular dementia. Modified versions of CSI–D and questionnaires were the most commonly used screening instruments. Diagnosis criteria for dementia are relatively adaptable to the community setting. However, most of the studies modified the screening instruments for the low literacy levels and local languages of the communities being examined, often involving the exclusion of sections of tests and adapting the diagnosis score. Many used an informant, often a family member, to gather a reliable history. Teams consisting of psychiatrists, clinical psychologists, neuropsychiatrists, neurologists, physicians or trained interviewers screened all of the populations.

### Determination of participants' age

As a rule, the age of each participant was verified in an official document, which in many cases involved birth certificates. However, in six of the studies there were substantial problems with age estimates in a considerable proportion of the participants, when official birth records were unavailable. In such cases, local historical landmark events were used to estimate the age of participants. This has been shown to be an accurate method of age estimation [12].

### Prevalence estimates

All 10 retained studies reported numerical estimates for dementia prevalence, with age breakdown of prevalence available from all studies but one [24]. No prevalence was recorded until the age of at least 50. Age groups 65–74 and 75–84 typically provided the most data points. **Figures 3**, **4** and **5** and **Tables 4**, **5** and **6** show that the prevalence of dementia grows from about 2% at the age of 60 to nearly 13% at the age of 90, with a trend to reach more than 25% at the age of 100. In comparison, Alzheimer disease has a prevalence of about 1% at 60 years, 2% at 70 years and slightly above 3% at 80 years, but then accelerates rapidly to more than 11% at the age of 90. In comparison, vascular dementia seems to reach about 1% of prevalence after the age of 60, but then the prevalence remains relatively stable and only begins to increase after the age of 85, to about 3%.

**Figure 6:**
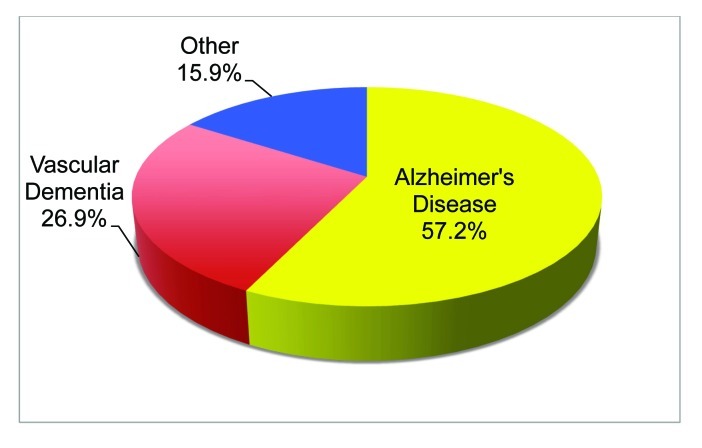
Proportional contribution of different types of dementia to the overall burden in Africa.

Although meta–analysis of the 10 retained studies in Africa would suggest that about 73% of the cases of dementia were Alzheimer disease and about 15% vascular dementia, those studies had a predominance of elderly people, and were therefore not representative of the current African population. The extrapolation of our findings to the current African population suggests that Alzheimer disease is currently responsible for 57.2% of all dementia cases in Africa, and vascular dementia for 26.9%, but with the former having a an increasing trend in relative importance (**Figure 6**).

We were also interested in investigating any differences in sex–specific prevalence of dementia. The only disease with sufficient evidence on gender breakdown was dementia, where 7 studies could be used. **Table 7** shows that females seem to have higher overall prevalence, particularly in very old age groups, although these data are based on relatively little information and surrounded with large uncertainty.

**Table 7:** A comparison of age–specific weighted mean prevalence of dementia in African men and women

Age group (years)	60–69	70–79	80+
Men:			
No. of data points	2	7	2
Mean age (years)	64.50	72.83	84.87
Weighted mean prevalence and standard error (in %)	1.93 (0.88)	2.60 (2.17)	16.83 (3.58)

Women:			
No. of data points	2	7	2
Mean age (years)	64.5	74.5	84.5
Weighted mean prevalence and standard error (in %)	1.35 (0.10)	5.22 (2.62)	19.68 (10.00)

Finally, the overall prevalence of dementia in adults older than 50 years in Africa was estimated to be about 2.4%, which translates to 2.76 million people living with a disease in 2010, and about 2.10 millions of them live in Sub–Saharan Africa. Further details are provided in **Table 8**.

**Table 8:** The estimates of the total number of cases of dementia, Alzheimer disease, vascular dementia and other forms of dementia in Africa in the year 2010 (figures in brackets: sub–Saharan Africa only)

Age group (years)	All dementia	Alzheimer disease	Vascular dementia	Other forms
0–54	308 513 (235 380)	96 608 (73 707)	185 615 (141 615)	26 290 (20 058)
55–59	352 334 (270 845)	130 713 (100 481)	128 145 (98 507)	93 476 (71 857)
60–64	388 370 (300 607)	170 352 (131 856)	101 769 (78 771)	116 250 (89 980)
65–69	401 815 (317 297)	208 666 (164 775)	89 998 (71 068)	103 151 (81 454)
70–74	399 054 (303 042)	245 459 (186 402)	84 044 (63 823)	69 550 (52 817)
75–79	342 986 (254 173)	249 838 (185 145)	69 972 (51 854)	23 176 (17 174)
80+	571 692 (413 919)	479 437 (347 124)	86 583 (62 688)	5673 (4107)
Total	2 764 764 (2 095 263)	1 581 073 (1 189 491)	746 126 (568 326)	437 565 (337 447)

### Risk factors for dementia

Eight papers reported on risk factors for dementia, in addition to prevalence estimates. The main findings are reported below and tabulated in Online Supplementary Document(Online Supplementary Document). All studies established increasing age as the strongest risk factor for the prevalence of dementia, with four studies also showing higher prevalence among females. Education is thought to be protective against dementia [31], so higher prevalence in women in Africa may reflect lower educational attainment and poorer socioeconomic status, another recognised risk factor for dementia [32]. The only study that carried out APOE genotyping reported higher prevalence of dementia in people with APOEϵ4 [27]. Poor nutritional status may also exacerbate the risk of developing dementia [18], and one study found a BMI<18.5 to be associated with dementia prevalence [11]. Another study found a lifetime of excessive alcohol consumption to double the risk dementia compared to those who were abstinent for life [29]. Hypertension and diabetes are emerging as significant endemic threats in Africa [8], which is notable as two of the studies reported cardiovascular problems as risk factors for vascular dementia: one study reported higher prevalence in urban areas [12], while another one reported higher prevalence in rural areas [29]. Other studies did not find study setting to represent a significant risk factor.

## DISCUSSION

This paper identified and analysed 10 studies reporting prevalence of dementia in African countries in order to provide an estimate for the overall prevalence of dementia in persons aged 50 years or more in Africa. It was conducted with an aim to provide recommendations for future policies and research.

### Findings

The overall prevalence of dementia in Africa in person aged 50 years or older, as modelled from 10 studies of sufficient quality and using UN's population estimates, was about 2.4%, which translates to 2.76 million people living with a disease in 2010. About 2.10 millions of them live in Sub–Saharan Africa. The highest reported prevalence was 10.1% across eight contiguous states in Nigeria and the lowest was 1.12% in Ibadan, Nigeria. The former value was from a study carried out in 2006, while the latter was in 1995, which may explain the large discrepancy between the two results from the same country. Age–specific analysis showed a general trend of an exponential increase in prevalence with age. The rough doubling of dementia prevalence every five years after the age of 65 is well documented in developed countries. The protective effect of oestrogen against dementia may account for lower prevalence of females in the 55–64 age group [18]. Alzheimer disease was found to be the most prevalent cause of dementia, followed by vascular dementia, and these findings match those of developed countries.

Previous estimates of dementia prevalence in Africa have been provided by the Alzheimer Disease International using a Delphi consensus study (for the years 2001 and 2020), and by the WHO (for the year 2010) [13,33]. The former predicted about 0.5 million affected persons in 2001 and 0.9 in 2020, although this estimate was restricted to Sub–Saharan Africa. It is apparent that the estimate based on expert opinion has already grossly underestimated the burden of this problem [33], which in our study already reached 2.1 million of sufferers in Sub–Saharan Africa in 2010. The WHO's estimate for 2010, of 1.9 million affected people in the population aged over–60, is much closer to our estimate which quotes the corresponding figure of about 2.1 million in persons over 60 years old. Our review estimates that at least 2.7 million people aged 50 years or more are living with dementia in Africa in 2010.

### Limitations

While this review aimed to provide the best possible estimate for the prevalence of dementia in Africa, there are features that may have limited the results. The overall data on dementia in Africa is insufficient, with only ten studies suitable for inclusion, and with the gender–aggregated and dementia subtypes prevalence available from only seven and six of the studies, respectively. All of the studies included data from >139 subjects, and one sample size was particularly large [25]. The similarity of prevalence values suggests that the range of sample sizes did not substantially skew the results. Methodologies did not vary too widely among studies, allowing for direct comparisons. The search was limited to 1980–present time, although most of the studies were active between 2002 and 2009. Considering the life expectancy at birth for Africans in 1980 was 48.5 years, it is unlikely that many, if any, prevalence studies for dementia were conducted before 1980 [34].

Case definitions of dementia, Alzheimer disease and vascular dementia were similar enough, with considerable overlap between them, for the results to be comparable. Misclassification bias may have occurred due to communication difficulties during assessment, especially as there is no definitive clinical diagnosis for dementia. It has been suggested that DSM–IV dementia criteria might underestimate dementia prevalence, especially in areas with low awareness of the emerging problem [8,35,36]. Another cause of underestimation is informant participation in the use of CSI–D, because of the cultural specific differences that prevent informants from disclosing information that could compromise their parents’ integrity [12], so that informants may give answers they think are acceptable rather than accurate [10]. Other notable aspects of the CSI–D instrument are that it should be independent of educational and cultural background of the subject, making it suitable where literacy level is low, and that it may have difficulty distinguishing depression from dementia [11].

All included studies were community–based, in order to avoid many biases present in facility–based studies or in other studies relevant only to specific population subgroups. Still, the quality of data is heavily reliant on the sensitivity and specificity of the screening methods and diagnostic criteria. Only three of the retained studies reported sensitivity and specificity of methods [11,12,29]. Standardisation of these tools is important to limit observational bias. Results may have depended on who the assessor was, with some assessors likely having more experience in dementia diagnosis than others, such as neurologists. Another concern is the focus of studies on certain regions in Africa. No studies were conducted in Eastern Africa, while six took place in Western Africa. Moreover, the general tendency of higher rates of illiteracy in rural areas, where many students leave school to work in the fields, may also potentially cause comparability issues.

### Awareness and diagnosis of dementia in developing countries

An ongoing debate on the lower prevalence of dementia in developing countries centres around the fact that the elderly in these regions are not expected to involve themselves in the instrumental activities of daily living (IADL) [31]. The living environment is often without sophisticated utilities, and therefore poses little cognitive challenge. Additionally, the social environment is one that protects the elderly rather than imposing tasks on them. This social environment which is relatively undemanding may mean that only those with severe dementia are likely to match social impairment criteria and receive diagnosis, thus under–estimating the true size of the problem [29]. Research and media attention in Africa is also mainly given to the diseases with higher case fatality, such as HIV and malaria. In comparison, dementia has a low mortality but high morbidity and disability. The general lack of awareness of the disease has important consequences: (i) help from medical services is not sought; (ii) no structured training exists on the recognition and management of dementia at any level of the health service; (iii) there is no constituency to place pressure on the government or policy makers to provide more responsive dementia care services; and (iv) families are the primary caregivers with little support from other individuals or agencies [15]. These features create a multi–faceted burden to the individual, their family and society, which has personal, emotional, financial and social aspects [17].

The affected person may experience ill health, disability, impaired quality of life and reduced life expectancy [14]. One study showed elevated odds for persons with dementia to be disabled in all areas of activities of daily living (ADL) and also having lower quality of life [29]. These findings correlate with the GBD reports, which indicate that dementia is one of the main causes of disability in later life [2]. Dementia can adversely affect communication, memory and mobility, creating dependence. It can affect sleep and mood, which can become a source of distress for caregivers, while creating feelings of loneliness and social isolation for the sufferers [15,37]. Due to the dependent nature of dementia, family and friends become a vital support network, but the reliability and universality of family care systems in LMIC are often overestimated [15,37]. Although there are many potential caregivers, this may not always translate into adequate care [31]. With the growing middle class, this situation is likely to change as family structures evolve toward western models and family support becomes less available [12]. Carers within family will find themselves in a difficult position, where they can either stop working in order to care for the affected person, thus losing the opportunity to earn income, or continuing with their work in parallel but reducing the outcomes for the affected. Caregiving is associated with high levels of psychological strain, and family caregivers have been shown to have increased levels of affective disorders such as depression and anxiety [13]. In addition, some studies reported a common misconception in many African settings that there is no treatment for dementia, and that it is a normal part of ageing [25]. This poses a problem, as dementia recognition would improve disease outcomes [15]. Diagnosis is crucial in that it provides access to treatment, care and support across the disease course. Earlier diagnosis is important for the affected person, as it allows people to plan ahead while they still have the capacity to do so; it also enables their caregivers to become better equipped and prepared [15]. Another consequence of the growing middle class is that a larger proportion of the population will be educated and have a higher awareness of the disease and possible treatments [7]. They are therefore more likely to seek better health care for themselves and their families. Combined with the substantial increases in dementia prevalence, these factors will place a considerable strain on already resource–scarce health services. Considering both this strain and the disintegration of multigenerational family structures, a purely health services response would be insufficient, and societal action is also needed.

Research evidence should inform the actions that are most needed, and each country should develop a priority research agenda. The course of dementia epidemics needs to be monitored nationally, focusing on changes in prevalence and incidence that might indicate success or failure of efforts to control it. Significantly more research is required to understand the causes of dementia, and how do lifestyle factors influence the risk [13]. Lack of consistency in the methodologies and case definitions of dementia between studies may hinder the comparability of the data. In this study, inconsistencies in reporting on the reviewed examinees were an important issue when standardising data. Standards to ensure synchronisation of methods would be helpful, such as types of assessor and screening measures. Suggestions of criteria for future studies are shown in Table 9. Diagnosis of dementia according to DSM criteria requires: (i) impairment in short– and long–term memory; (ii) impairment in abstract thinking or judgement, or impairment of higher cortical functions or personality changes; and (iii) evidence that the cognitive disturbances resulting from (i) and (ii) significantly interfere with work, usual social activities or relationships with others [38]. DSM criteria were chosen over ICD criteria because (i) they are defined more precisely; and (ii) ICD criteria are more dependent on informants, making them harder to apply to a community setting [39].

**Table 9:** Recommended criteria for future studies of dementia in the community

Criteria	Minimum required standard
**Case definition**	DSM criteria. Screening measures: SSQ, CSI–D & FCA. Complication screening measures: Goldberg’s Anxiety and Depression Scale, psychosocial factors according to Persson and Skoog, WHO–QOL–BREF.
**Methodology**	Two–phase dementia diagnosis study. Door–to–door survey of all households. Interview prior to start of study should obtain demographic and risk factors.
**Assessor**	Specially trained investigator; training includes symptom recognition, physical examination and patient communication skills.
**Population**	All adults included, representative of community population. Replacement subjects matched to drop–outs. Prevalence and incidence data grouped by age (18–49 y, by 10 y thereafter), sex (M/F) and subtype (Alzheimer disease, vascular dementia, Lewy body dementia, frontotemporal dementia and others).
**Sample size**	>500 subjects
**Duration**	>1 year

FCA – Family Caregiver Alliance, SSQ – Speech, Spatial and Qualities of Hearing Scale, CSI–D – Community Screening Interview for Dementia, WHO–QOL–BREF – WHO Quality of Life–BREF

Although two–phase diagnosis studies are thought to increase the rates of attrition before clinical assessment, some authors found them to be feasible in African countries due to high participation rates [8]. Trained investigators should be used, so that any additional burden on practicing physicians is avoided. All adults in the population aged 50 or older should be screened, to prevent missing a diagnosis of early–onset Alzheimer disease.

There are considerable further obstacles that should be addressed by policies to control dementia in Africa: (i) generally low priority of mental and neurological disorders; (ii) poor awareness and understanding of those diseases; and (iii) lack of infrastructure and resources [13]. Evidence from this review has shown that dementia prevalence in Africa is increasing towards the levels that are present in developed countries, and this trend will continue to increase at a faster rate than anywhere else in the world. Resources need to be set aside to tackle this growing problem at national, local, family and personal levels. Attention must be given to reducing the burden of disease through timely and appropriate diagnosis and treatment, symptom management and reduction in exposure to risk factors. Public campaigns will need to be launched to fight common misconceptions regarding dementia, such as that: (i) it is not very common; (ii) it is a normal part of ageing; (iii) there is nothing that can be done to help; (iv) it is better not to know about it; and that (v) it is the responsibility of the family to provide care. The varying presentation and impact of dementia make it challenging to alter the social and cultural determinants of care–seeking behaviour. Many of the risk factors identified in this review, such as age and sex, require a grass–roots approach in order to raise awareness of and educate the general public about dementia. It is important that people’s cultures and beliefs are considered when developing awareness–raising campaigns. Other risk factors are linked to the structural determinants of disease and require multi–sectoral action. It is important that health care workers are well trained in case management of dementia, with the ability to efficiently assess, diagnose, treat or refer and educate patients and their caregivers. This approach should limit distress in the affected person, reduce strain and build the confidence of carers, and reduce unnecessary consultations.

Strategies to raise awareness of dementia and improve the chance of early diagnosis include: (i) practice–based educational programmes in primary care; (ii) the introduction of accessible diagnostic and early stage dementia care services (eg, memory clinics); and (iii) promoting effective interaction between different components of the health system [16]. With the growing middle class in Africa, established cultural norms of care and social protection through extended family networks are no longer sustainable. A key policy priority therefore is to plan for the long–term care of people with dementia. In addition, more research needs to be commissioned and funded in order to improve understanding of the different social and environmental risk factors.

### Conclusion

The number of elderly people in Africa is increasing rapidly, with an accelerating number of the persons affected by dementia. Although the epidemiological literature on dementia in Africa is very limited, our estimates of the number of cases living with dementia in Africa suggest that this condition should already be considered a public health priority. The analysis of the study design of the available research indicated that the prevalence is likely to be underestimated, and that the true figures are likely to be considerably higher. The evidence base of epidemiological data of dementia must be improved to fully understand the burden and its impacts on individuals, families and societies. More research is needed in the entire Africa, but particularly in the southern and eastern regions. Consistency in case definition and methodologies are vital in creating a reliable estimate. There is also a need for age– and sex–specific information in order to refine the existing burden estimates and provide specific targets for health programmes. Other research priorities include multiple collaborative community–based studies in the adult population and investigations of lifestyle factors that influence development of the disease. Policy recommendations include raising awareness of the disease through educational programmes in primary care, the introduction of diagnostic and early stage dementia care services in the community, and collaboration between different components of the health system. Planning for the long–term care of sufferers and their families is encouraged in light of the growing middle class in Africa.

## References

[1] World Health Organization. Global status report on noncommunicable diseases. Geneva: WHO, 2010.

[2] World Health Organization. The Global Burden of Disease: 2004 update. Geneva: WHO, 2008.

[3] Reardon S (2011). Meeting brings attention but little action on chronic diseases.. Science.

[4] United Nations. World Population Ageing 2009. New York:United Nations, 2009.

[5] United Nations. World Population Prospects, the 2010 Revision. UNdata 2011. [Online] Available at: http://data.un.org/Data.aspx?d=PopDiv&f=variableID%3A68 Accessed: 24 April 2012.

[6] Daar AS, Singer PA, Persad DL, Pramming SK, Matthews DR, Beaglehole R (2007). Grand challenges in chronic non-communicable diseases.. Nature.

[7] African Development Bank Group. The middle of the pyramid: Dynamics of the middle class in Africa. Market Brief, 20 April 2011 [Online]. Available at: http://www.afdb.org/fileadmin/uploads/afdb/Documents/Publications/The%20Middle%20of%20the%20Pyramid_The%20Middle%20of%20the%20Pyramid.pdf Accessed: 24 April 2012.

[8] Guerchet M, M’belesso P, Mouanga AM, Bandzouzi B, Tabo A, Houinato DS (2010). Prevalence of dementia in elderly living in two cities of central Africa: The EDAC survey.. Dement Geriatr Cogn Disord.

[9] Beaglehole R, Bonita R, Alleyne G, Horton R, Liming L, Lincoln P (2011). UN high-level meeting on non-communicable diseases: addressing four questions.. Lancet.

[10] Ineichen B (2000). The epidemiology of dementia in Africa: a review.. Soc Sci Med.

[11] Ochayi B, Thacher TD (2006). Risk factors for dementia in central Nigeria.. Aging Ment Health.

[12] Parad’so MN, Guerchet M, Saizonou J, Cowppli-Bony P, Mouanga AM, Nubukpo P (2011). Prevalence of dementia among elderly people living in Cotonou, an urban area of Benin (West Africa).. Neuroepidemiology.

[13] World Health Organization. Dementia: a public health priority. Geneva: WHO, 2012.

[14] Alzheimer’s Disease International. World Alzheimer Report 2009. Illinois, USA: ADI, 2009.

[15] World Health Organization. Neurological disorders: public health challenges. Geneva: WHO, 2006.

[16] Alzheimer’s Disease International. World Alzheimer Report 2011. Illinois, USA: ADI, 2011.

[17] Alzheimer’s Disease International. World Alzheimer Report 2010. Illinois, USA: ADI, 2010.

[18] Alzheimer Scotland. Risk factors in dementia. Edinburgh: Alzheimer Scotland, 2011.

[19] Brookmeyer R, Johnson E, Ziegler-Graham K, Arrighi HM (2007). Forecasting the global burden of Alzheimer’s disease.. Alzheimers Dement.

[20] Beaglehole R, Yach D (2003). Globalisation and the prevention and control of non-communicable disease: the neglected chronic diseases of adults.. Lancet.

[21] Beaglehole R, Bonita R, Horton R, Adams C, Alleyne G, Asaria P (2011). Priority actions for the non-communicable disease crisis.. Lancet.

[22] Kalaria RN, Maestre GE, Arizaga R, Friedland RP, Galasko D, Hall K (2008). Alzheimer’s disease and vascular dementia in developing countries: prevalence, management, and risk factors.. Lancet Neurol.

[23] Prince M, Patel V, Saxena S, Maj M, Maselko J, Phillips MR (2007). No health without mental health.. Lancet.

[24] Ben-Arie O, Swartz L, Teggin AF, Elk R (1983). The coloured elderly in Cape Town – a psychosocial, psychiatric and medical community survey: Part II. Prevalence of psychiatric disorders.. S Afr Med J.

[25] El Tallawy HNA, Farghaly WMA, Metwaly NA, Rageh TA, Shehata GA, Elftoh NA (2010). Door-to-door survey of major neurological disorders in Al Kharga district, New Valley, Egypt: methodological aspects.. Neuroepidemiology.

[26] Farrag A, Farwiz HM, Khedr EH, Mahfouz RM, Omran SM (1998). Prevalence of Alzheimer's disease and other dementing disorders: Assiut-Upper Egypt study.. Dement Geriatr Cogn Disord.

[27] Guerchet M, Houinato D, Paraiso MN, von Ahsen N, Nubukpo P, Otto M (2009). Cognitive impairment and dementia in elderly people living in rural Benin, West Africa.. Dement Geriatr Cogn Disord.

[28] Guerchet M, M'belesso P, Mouanga AM, Bandzouzi B, Tabo A, Houinato DS (2010). Prevalence of dementia in elderly living in two cities of Central Africa: the EDAC survey.. Dement Geriatr Cogn Disord.

[29] Gureje O, Ogunniyi A, Kola L (2006). The profile and impact of probably dementia in a sub-Saharan African community: results from the Ibadan Study of Aging.. J Psychosom Res.

[30] Hendrie HC, Osuntokun BO, Hall KS, Ogunniyi AO, Hui SL, Unverzagt FW (1995). Prevalence of Alzheimer’s disease and dementia in two communities: Nigerian Africans and African Americans.. Am J Psychiatry.

[31] Yusuf AJ, Baiyewu O, Sheikh TL, Shehu AU (2011). Prevalence of dementia and dementia subtypes among community-dwelling elderly people in northern Nigeria.. Int Psychogeriatr.

[32] Gureje O, Ogunniyi A, Kola L, Abiona T (2011). Incidence and risk factors for dementia in the Ibadan Study of Aging.. J Am Geriatr Soc.

[33] Ferri CP, Prince M, Brayne C, Brodaty H, Fratiglioni L, Ganguli M (2005). Global prevalence of dementia: a Delphi consensus study.. Lancet.

[34] United Nations. World Population Prospects, the 2010 Revision. 28 June 2011 [Online]. Available at: http://esa.un.org/unpd/wpp/Excel-Data/mortality.htm Accessed: 19 April 2012.

[35] El Tallawy HNA, Farghaly WMA, Rageh TA, Shehata GA, Metwaly NA, Elftoh NA (2010). Epidemiology of major neurological disorders project in Al Kharga district, New Valley, Egypt.. Neuroepidemiology.

[36] Prince M, Acosta D, Chiu H, Scazufca M, Verghese M (2003). Dementia diagnosis in developing countries: a cross-cultural validation study.. Lancet.

[37] Elk R, Swartz L, Gillis LS (1983). The Coloured elderly in Cape Town – a psychosocial, psychiatric and medical community survey. Part I. Introduction and psychosocial data.. S Afr Med J.

[38] Riedel-Heller SG, Busse A, Aurich C, Matschinger H, Angermeyer MC (2001). Prevalence of dementia according to DSM-III-R and ICD-10: Results of the Leipzig Longitudinal Study of the Aged (LEILA75+). Part I.. Br J Psychiatry.

[39] Naik M, Nygaard HA (2008). Diagnosing dementia – ICD-10 not so bad after all: a comparison between dementia criterion according to DSM-IV and ICD-10.. Int J Geriatr Psychiatry.

