# George-Carey R, Adeloye A, Chan KY, Paul A, Kolčić I, Campbell H, Rudan I. An estimate of the prevalence of dementia in Africa: A systematic analysis. *J Glob Health* 2013;2:020401corr1.

**DOI:** 10.7189/jogh.020401corr1

**Published:** 2012-12

The authors informed the publisher that the term Democratic Republic of Congo (capital: Kinshasa) was used instead of the correct name of the country – Republic of Congo (capital: Brazzaville) in Figure 2, Tables 2 and 3 and the text. The author and the publisher apologise for this error. We have since corrected the online version of the article.

