# Supplementary material for: An estimate of the prevalence of dementia in Africa: A systematic analysis
Source: J Glob Health. 2012 Dec;2(2):020401. doi: 10.7189/jogh.02.020401 (PMC3529309; doi:10.7189/jogh.02.020401)
Supplement: Online Supplementary Document [file jogh-02-020401-s001.pdf]

Online Supplementary Document

George-Carey et al. An estimate of the prevalence of dementia in Africa: A systematic analysis

Journal of Global Health 2012;2:020401

Table w1. Prevalence of dementia in retained studies

African region	Article	Country	Method	Prevalence (%)	95% confidence intervals
Northern	El Tallawy (2010)	Egypt	SSQ, FCA	2.20	n/a
	Farrag (1998)	Egypt	SSQ, MMSE, FCA, Lab tests	4.50	<1
Western	Guerchet (2009)	Benin	SSQ, CSI-D, FCA	2.60	1.1 – 3.8
	Gureje (2006)	Nigeria	10WDRT	10.10	8.6 – 11.8
	Hendrie (1995)	Nigeria	SSQ, CSI-D, CERAD	2.29	0.0 – 2.24
	Ochayi (2006)	Nigeria	CSI-D	6.40	3.8 – 9.9
	Paraiso (2011)	Benin	SSQ, CSI-D, FCA	3.70	2.6 – 4.8
	Yusuf (2011)	Nigeria	SSQ, CSI-D, CERAD, BDS, SDT	3.79	1.0 – 4.58
Central	Guerchet (2010)	Central African Republic	SSQ, CSI-D, 5WT, FCA	8.10	5.8 – 10.8
		Democratic Republic of the Congo	SSQ, CSI-D, 5WT, FCA	6.70	4.7 – 9.2
Southern	Ben-Arie (1983)	South Africa	MMSE, PSE, FCA	8.60	n/a

MMSE – Mini Mental State Examination, PSE – portal-systemic shunt encephalopahty, FCA – Family Caregive Alliance, SSQ – Speech, Spatial and Qualities of Hearing Scale, CSI-D – CommunityScreening Interview for Dementia, 5WT – Five Word Test, 10WDRT – Ten Word Test,CERAD – Consortium to Establish a Registry for Alzheimer Disease, SDT – sibship disequilibrium test, BDS – Blessed Dementia Scale, IADL – Instrumental Activities of Daily Living

Table w2. Prevalence (%) of dementia subtypes in retained studies

Article	Country	Minimum age	Sample size	Subtypes	Prevalence (%)
Farrag (1998)	Egypt	60 years	2000	Alzheimer’s disease	2.20
				Vascular dementia	0.45
				Other	0.50
Hendrie (1995)	Nigeria	65 years	2494	Alzheimer’s disease	0.72
				Vascular dementia	0.32
				Other	0.04
Guerchet (2009)	Benin	65 years	502	Alzheimer’s disease	2.20
				Vascular dementia	0.20
				Other	0.20
Guerchet (2010)	Central African Republic	65 years	509	Alzheimer’s disease	6.65
				Vascular dementia	1.41
Guerchet (2010)	Democratic Republic of the Congo	65 years	546	Alzheimer’s disease	4.62
				Vascular dementia	2.12
Paraiso (2011)	Benin	65 years	1139	Alzheimer’s disease	2.81
				Vascular dementia	0.80
				Other	0.09
Yusuf (2011)	Nigeria	65 years	322	Alzheimer’s disease	1.86
				Vascular dementia	0.62
				Other	0.31

Table w3. Full study characteristics

Reference	Country	City/Area	Period of study	Setting	Age group	Population characteristics	Study size	Assessor
Ben-Arie (1983)	South Africa	Cape Town	May – June 1982	Urban	65+	Systematic sampling technique involving houses aged 65+ years	139	6 trained interviewers, 15 medical practitioners and 10 psychiatrists
El Tallawy (2010)	Egypt	Al Kharga District, New Valley	June 2005 – May 2009	Urban and Rural	50+	Eligible inhabitants who had been living in the area for at least 6 months at time of study	62,583	Neurologists
Farrag (1998)	Egypt	Assiut-Upper	May 1993 – August 1994	Urban and Rural	60+	Random sample of all household aged 60+	2,000	Research team chaired by neuropsychiatrist
Guerchet (2009)	Benin	Djidja	March – April 2007	Rural	65+	Door-to-door screening in 7 randomly selected neighboured villages	502	Trained interviewers, neurologist
Guerchet (2010)	Central African Republic	Bangui	September 2008 – March 2009	Urban	65+	Door-to-door screening	1,016	Trained interviewers, local neurologists and psychiatrists and an epidemiologist
	Democratic Republic of the Congo	Brazzaville						

Gureje (2006)	Nigeria	Lagos, Ugun, Osun, Oyo, Ondo, Ekiti, Kog, Kwara	November 2003 – August 2004	Urban	65+	Four-stage area probability sampling of households	2,152	24 trained interviewers
Hendrie (1995)	Nigeria	Ibadan	May 1992 – March 1994	Urban	65+	Door-to-door screening in a geographically defined area	2,494	Trained interviewers
Ochayi (2006)	Nigeria	Jos South Local Government Area	January – December 2002	Rural	65+	Stratified random sampling technique of one street form each ward	280	Trained interviewers
Paraiso (2011)	Benin	Cotonou	May – September 2008	Urban	65+	People aged 65+ who had lived in Cotonou for at least 5 years	1,139	10 investigators, neurologist, 2 physicians
Yusuf (2011)	Nigeria	Zaria	March – October 2007	Urban	65+	Systematic sampling technique	322	Panel of 6 physicians (2 translators)

Table w4. Risk factors for dementia

Article	Date	Minimum age	Risk factors
Ben-Arie (1983)	May – June 1982	65 years	Increasing age, hypertension
El Tallawy (2010)	June 2005 – May 2009	50 years	n/a
Farrag (1998)	May 1993 – August 1994	60 years	Female sex, increasing age (especially 75+ years)
Guerchet (2009)	March – April 2007	65 years	Increasing age, APOEε4, depression
Guerchet (2010)	September 2008 – March 2009	65 years	Increasing age, CVD risk factors, hypertension, psychosocial risk factors
Gureje (2006)	November 2003 – August 2004	65 years	Female sex, increasing age (especially 80+ years), lifetime history of alcohol misuse
Hendrie (1995)	May 1992 – March 1994	65 years	n/a
Ochayi (2006)	January – December 2002	65 years	Female sex, increasing age, low education level, low BMI
Paraiso (2011)	May – September 2008	65 years	Female sex, increasing age, urban setting, hypertension, social isolation
Yusuf (2011)	March – October 2007	65 years	Female sex, increasing age (especially 80+ years), < 6 years of local education
